# Fast and Accurate Numerical Integration of the Langevin Equation with Multiplicative Gaussian White Noise

**DOI:** 10.3390/e26100879

**Published:** 2024-10-20

**Authors:** Mykhaylo Evstigneev, Deniz Kacmazer

**Affiliations:** Department of Physics and Physical Oceanography, Memorial University of Newfoundland, St. John’s, NL A1B 3X7, Canada; dkacmazer@mun.ca

**Keywords:** Langevin equation, multiplicative noise, diffusion, computational physics

## Abstract

A univariate stochastic system driven by multiplicative Gaussian white noise is considered. The standard method for simulating its Langevin equation of motion involves incrementing the system’s state variable by a biased Gaussian random number at each time step. It is shown that the efficiency of such simulations can be significantly enhanced by incorporating the skewness of the distribution of the updated state variable. A new algorithm based on this principle is introduced, and its superior performance is demonstrated using a model of free diffusion of a Brownian particle with a friction coefficient that decreases exponentially with the kinetic energy. The proposed simulation technique proves to be accurate over time steps that are an order of magnitude longer than those required by standard algorithms. The model used to test the new numerical technique is known to exhibit a transition from normal diffusion to superdiffusion as the environmental temperature rises above a certain critical value. A simple empirical formula for the time-dependent diffusion coefficient, which covers both diffusion regimes, is introduced, and its accuracy is confirmed through comparison with the simulation results.

## 1. Introduction

The Langevin equation (LE) provides a computationally efficient method for modeling the dynamics of a system interacting with a heat bath, where the heat bath’s uncontrollable degrees of freedom are accounted for as a dissipative and a random force. If the heat bath responds to changes in the system’s state much faster than these changes occur, the dissipative force depends only on the system’s current state rather than its previous evolution; additionally, the noise in the LE can be approximated as Gaussian and white [1,2].

The most general form of the Langevin equation features a multiplicative noise term, while the version with additive noise [3] represents an important and arguably the most extensively studied special case. The LE with multiplicative noise is particularly relevant for diffusion problems [4,5,6,7], where the effect of the medium in which a Brownian particle resides depends on the particle’s energy.

Numerical simulations of the LE are widely performed across various disciplines, including physics, mathematics, chemistry, biology, and finance [1,2,8]. Over the years, efficient algorithms have been developed for this purpose [8,9,10,11,12,13,14,15,16,17,18,19,20]. In these simulations, time is discretized into small intervals, and the system’s state is updated at each step according to a rule that invariably involves incrementing the system’s state by a Gaussian random number [8,9,10,11,12,13,14,15,16,17,18,19,20]. The choice of the simulation time step is crucial: a step that is too large sacrifices accuracy, while one that is too small can make the simulations excessively time-consuming.

This paper examines a univariate Langevin equation with multiplicative Gaussian white noise. The standard second-order numerical scheme for propagating the system’s state under such dynamics, introduced in Ref. [10], is revisited. We demonstrate that its computational efficiency can be significantly enhanced by modifying the update process for the system’s state variable, allowing the simulation time step to be increased by as much as an order of magnitude without losing the accuracy of the numerical procedure.

The organization of this paper is as follows: In the next section, we formulate the relevant LE and describe its standard simulation method. In Section 3, we introduce an alternative scheme. Subsequently, we demonstrate its superior performance using a model of one-dimensional Brownian motion with velocity-dependent friction [7]. This model is further explored with the help of our new numerical algorithm in Section 4. An empirical formula that describes the time-dependent diffusion coefficient over many time scales is introduced and tested against the simulation results. This formula applies in both regular diffusion and superdiffusive regimes of motion. Finally, potential avenues for further development of the proposed scheme are briefly discussed in the concluding section (Section 5).

## 2. Standard Integration of the Langevin Equation

Here, we consider a system whose state is described by a single variable *z* that changes in time according to the Langevin equation (LE) with multiplicative noise [2]:

(1) $$\dot{z}=h\left(z\right)+g\left(z\right)\;\xi \left(t\right)\;.$$

The properties of Gaussian white noise ξ(t) are

(2) $$\langle \xi \left(t\right)\rangle =0\;,\;\;\langle \xi \left(t\right),\xi \left(t^{\prime }\right)\rangle =\delta \left(t-t^{\prime }\right)\;,$$

and the last term in Equation (1) is interpreted in the Stratonovich sense [2,21].

The standard procedure [10,15,16,19] for numerically integrating Equation (1) involves discretizing time into small steps of size Δt and updating the state variable to a new value $z^{\prime }$ according to the following rule:

(3) $$z^{\prime }=z+\bar{\Delta z}+\sqrt{\mu_{2}}\;\Gamma \;.$$

Here, the average displacement over the time step Δt is given by [21]

(4) $$\bar{\Delta z}=H\;\Delta t+\frac{\Delta t^{2}}{2}\left(G\;\frac{d^{2}H}{dz^{2}}+H\;\frac{dH}{dz}\right)\;,$$

with

(5) $$G\left(z\right)=\frac{g^{2}\left(z\right)}{2}\;,\;\;H\left(z\right)=h\left(z\right)+\frac{g(z)}{2}\;\frac{dg(z)}{dz}\;.$$

Additionally, Γ is a Gaussian random variable with unit variance, distributed as

(6) $$W\left(\Gamma \right)=\frac{1}{\sqrt{2\pi }}\;e^{-\Gamma^{2}/2}\;,$$

and the dispersion of $z^{\prime }$ around $z+\bar{\Delta z}$ is expressed as

(7) $$\mu_{2}\left(z\right)=2G\;\Delta t+\left(2G\;\frac{dH}{dz}+G\;\frac{d^{2}G}{dz^{2}}+H\;\frac{dG}{dz}\right)\;\Delta t^{2}\;.$$

The derivation of the rule (3) is typically performed by integrating the Langevin equation [10,16] using the Stratonovich interpretation for integrals involving the noise ξ(t). In the next section (Section 3), we present an alternative derivation of this prescription from a more general $z\to z^{\prime }$ update rule.

## 3. Improved Second-Order Integration Scheme

### 3.1. Description of the Method

We begin by recalling the observation made in Ref. [10] that including the higher-order terms in the average displacement (4) and dispersion (7) does not improve the accuracy of the integration scheme (3). We propose the following interpretation of this observation: the distribution of the displacements

(8) $$\Delta z=z^{\prime }-z$$

can only be reasonably approximated as Gaussian when the time step Δt is sufficiently small. For larger values of Δt, the actual distribution of Δz deviates from a Gaussian profile and may even become asymmetric. Consequently, the numerical scheme (3), which relies on approximating the distribution of Δz as Gaussian, will inevitably introduce a systematic error, regardless of how precisely the mean and dispersion of this distribution are determined.

A possible solution to this limitation is to replace the Gaussian distribution (6) with a non-Gaussian alternative. Indeed, earlier work [10,11] has suggested that such a replacement is feasible. However, a recent study [20] provides a strong argument that W(Γ) should remain Gaussian if one wishes to keep the time step Δt reasonably large.

To preserve the Gaussian nature of the random variable Γ while allowing the displacement Δz to follow a non-Gaussian distribution, we generalize the update rule as follows:

(9) $$\Delta z=a+b\;\Gamma +c\;\Gamma^{2}\;.$$

The coefficients *a*, *b*, and *c* can be determined from the first three moments of Δz as follows:

(10) $$\begin{array}{cc} \bar{\Delta z} & =a+c\;,\\ \bar{\Delta z^{2}} & =a^{2}+2ac+3c^{2}+b^{2}={\bar{\Delta z}}^{2}+2c^{2}+b^{2}\;,\\ \bar{\Delta z^{3}} & =a^{3}+3a^{2}c+9ac^{2}+15c^{3}+3ab^{2}+9b^{2}c\\ \; & ={\bar{\Delta z}}^{3}+3\;\left(\bar{\Delta z}+2c\right)\;\left(\bar{\Delta z^{2}}-{\bar{\Delta z}}^{2}\right)-4c^{3}\;, \end{array}$$

as can be obtained by averaging various powers of the right-hand side of Equation (9) with respect to Γ according to the distribution (6).

To find the coefficients *a*, *b*, and *c* in Equation (9), it is convenient to use the central moments of Δz:

(11) $$\begin{array}{cc} \; & \mu_{2}=\bar{{\left(\Delta z-\bar{\Delta z}\right)}^{2}}=\bar{\Delta z^{2}}-{\bar{\Delta z}}^{2}\;,\\ \; & \mu_{3}=\bar{{\left(\Delta z-\bar{\Delta z}\right)}^{3}}=\bar{\Delta z^{3}}-{\bar{\Delta z}}^{3}-3\mu_{2}\;\bar{\Delta z}\;, \end{array}$$

in terms of which the equation for *c* is written as

(12) $$c^{3}-\frac{3}{2}\;\mu_{2}c+\frac{1}{4}\;\mu_{3}=0\;.$$

This is a suppressed cubic equation. If $|\mu_{3}|\;>2\sqrt{2}\;\mu_{2}^{3/2}$, it has a single real-valued root; otherwise, it has three real roots. We are interested in the one that vanishes as $\mu_{3}\to 0$.

Although an exact solution of a cubic equation is known, we opt for a simpler approach, where the central moments $\mu_{2}$ and $\mu_{3}$ are expressed as power series in Δt, truncated at a desired order, which we choose to be $\Delta t^{2}$. Likewise, the unknown parameter *c* is represented as a power series

(13) $$c=c_{1}\;\Delta t+c_{2}\;\Delta t^{2}+\ldots$$

with the coefficients $c_{1}$, $c_{2}$, …expressed in terms of the expansion coefficients of the central moments $\mu_{2}$, $\mu_{3}$ by equating equal powers of Δt in Equation (12). Once the parameter *c* is obtained, the values of *a* and *b* are immediately found from the first two in Equation (10):

(14) $$b=\sqrt{\mu_{2}-2c^{2}}\;,\;\;a=\bar{\Delta z}-c\;.$$

Hence, our next task is to find $\mu_{2}$ and $\mu_{3}$ to the second order in Δt.

### 3.2. Calculation of the Moments

Equivalent to the LE (1) for the stochastic trajectory z(t) is the Fokker–Planck equation (FPE) for the transition probability $P(z^{\prime },t|z)$ from the initial state *z* to the final step $z^{\prime }$ [2]:

(15) $$\begin{array}{cc} \dot{P}\left(z^{\prime },t|z\right)=\hat{L}\left(z^{\prime }\right)\;P & =\frac{\partial }{\partial z^{\prime }}\left(\frac{g^{2}\left(z^{\prime }\right)}{2}\;\frac{\partial P}{\partial z^{\prime }}-\left(h\left(z^{\prime }\right)-\frac{g(z^{\prime })}{2}\;\frac{dg(z^{\prime })}{dz^{\prime }}\right)P\right) \end{array}$$

with the initial condition

(16) $$P\left(z^{\prime },0|z\right)=\delta \left(z^{\prime }-z\right)\;.$$

Given the initial state *z* at time *t*, the formal solution of (15) at the next time step t+Δt is

(17) $$P\left(z^{\prime },\Delta t|z\right)=e^{\hat{L}\left(z^{\prime }\right)\;\Delta t}\;\delta \left(z^{\prime }-z\right)\;.$$

We define the expectation value of an arbitrary function $f(z^{\prime })$ at time t+Δt, given the initial state *z* at time *t*, as

(18) $$\bar{f}\left(z\right)=\int dz^{\prime }\;f\left(z^{\prime }\right)\;P\left(z^{\prime },\Delta t|z\right)\;.$$

Note that $\bar{f}$ depends on the initial state *z*, not on the final state $z^{\prime }$.

Incorporating Equation (17) into this definition, we have

(19) $$\bar{f}\left(z\right)=\int dz^{\prime }\;f\left(z^{\prime }\right)\;e^{\hat{L}\left(z^{\prime }\right)\;\Delta t}\;\delta \left(z^{\prime }-z\right)=\int dz^{\prime }\;\left(e^{{\hat{L}}^{†}\left(z^{\prime }\right)\;\Delta t}\;f\left(z^{\prime }\right)\right)\;\delta \left(z^{\prime }-z\right)=e^{{\hat{L}}^{†}\left(z\right)\;\Delta t}\;f\left(z\right)\;,$$

where the adjoint Fokker–Planck operator is

(20) $${\hat{L}}^{†}\left(z\right)\;f\left(z\right)=\frac{d}{dz}\left(G\;\frac{df}{dz}\right)+\left(h-\frac{1}{2}\;g\;\frac{dg}{dz}\right)\;\frac{df}{dz}=G\;\frac{d^{2}f}{dz^{2}}+H\;\frac{df}{dz}$$

and its exponentiation is understood in the sense of a Taylor series.

Taking the function $f(z^{\prime })$ to be ${\left(z^{\prime }-z\right)}^{n}$ with n=1,2,3, we obtain the displacement moments up to the terms of the order of $\Delta t^{3}$:

(21) $$\begin{array}{cc} \bar{\Delta z} & =\left(e^{{\hat{L}}^{†}\;\Delta t}-1\right)\;z=\Delta t\;{\hat{L}}^{†}\;z+\frac{\Delta t^{2}}{2}\;{\left({\hat{L}}^{†}\right)}^{2}\;z=\Delta t\;H+\frac{\Delta t^{2}}{2}\;\left(G\;\frac{d^{2}H}{dz^{2}}+H\;\frac{dH}{dz}\right)\;,\\ \bar{\Delta z^{2}} & =\left(e^{{\hat{L}}^{†}\;\Delta t}+1\right)\;z^{2}-2z\;e^{{\hat{L}}^{†}\;\Delta t}\;z=\Delta t\;\left({\hat{L}}^{†}\;z^{2}-2z\;{\hat{L}}^{†}\;z\right)+\frac{\Delta t^{2}}{2}\;\left({\left({\hat{L}}^{†}\right)}^{2}\;z^{2}-2z\;{\left({\hat{L}}^{†}\right)}^{2}\;z\right)\;,\\ \; & =2G\;\Delta t+\Delta t^{2}\left(H^{2}+2G\;\frac{dH}{dz}+G\;\frac{d^{2}G}{dz^{2}}+H\;\frac{dG}{dz}\right)\;,\\ \bar{\Delta z^{3}} & =\left(e^{{\hat{L}}^{†}\;\Delta t}-1\right)\;z^{3}-3z\;\left(e^{{\hat{L}}^{†}\;\Delta t}\;z^{2}-z\;e^{{\hat{L}}^{†}\;\Delta t}\;z\right)=\Delta t\;\left({\hat{L}}^{†}z^{3}-3z\;{\hat{L}}^{†}\;z^{2}+3z^{2}\;{\hat{L}}^{†}z\right)\\ \; & +\frac{\Delta t^{2}}{2}\;\left({\left({\hat{L}}^{†}\right)}^{2}z^{3}-3z\;{\left({\hat{L}}^{†}\right)}^{2}\;z^{2}+3z^{2}\;{\left({\hat{L}}^{†}\right)}^{2}z\right)=6G\;\left(H+\frac{dG}{dz}\right)\;\Delta t^{2}\;. \end{array}$$

It is seen that the average displacement is indeed given by Equation (4), and the displacement second moment is given by Equation (7). We also obtain the third central moment up to $O(\Delta t^{3})$:

(22) $$\mu_{3}=6\;\Delta t^{2}\;G\;\frac{dG}{dz}\;.$$

Note that the third central moment $\mu_{3}$ differs from zero only when noise is multiplicative; for additive noise, the function G(z) is in fact a constant, and $\mu_{3}=0$. This also means that the skewness of the distribution of the updated state variable is only important when the noise is multiplicative, at least in the second order.

Incorporating the expressions for the central moments $\mu_{2}$ and $\mu_{3}$ into Equation (12), we obtain the coefficients from Equation (13):

(23) $$\begin{array}{cc} \; & c_{1}=\frac{1}{2}\;\frac{dG}{dz}\;,\\ \; & c_{2}=-\frac{1}{2}\;\frac{dG}{dz}\;\left(\frac{dH}{dz}+\frac{1}{2}\;\frac{d^{2}G}{dz^{2}}+\frac{H}{2}\;\frac{d\;\ln G}{dz}\right)+\frac{1}{24}\;G^{2}\;{\left(\frac{d\ln G}{dz}\right)}^{3}\;, \end{array}$$

The remaining coefficients *a* and *b* from Equation (9) are immediately obtained using Equation (14).

## 4. Case Study: Free Diffusion with Velocity-Dependent Friction

### 4.1. The Model

To demonstrate the superior performance of our numerical integration scheme from Section 3 relative to the standard one, we focus on a model of one-dimensional diffusion of a Brownian particle that experiences Stokes-like friction. It is assumed that the respective friction coefficient γ(v) decreases exponentially with the particle’s kinetic energy [7]:

(24) $$m\dot{v}=-\gamma \left(v\right)\;v+g\left(v\right)\;\xi \left(t\right)\;,\;\;\dot{x}=v\;,\;\;\gamma \left(v\right)=\gamma_{0}\;e^{-v^{2}/u^{2}}\;,$$

where *m* is the particle mass and $\gamma_{0}$ and *u* are constants.

The noise-coupling function g(v) is uniquely related to the friction coefficient γ(v) by the fluctuation–dissipation theorem of the second kind generalized to the multiplicative noise case [6,7]:

(25) $$g^{2}\left(v\right)=2m\;e^{mv^{2}/T}\;\int_{v^{2}}^{\infty }dy\;\gamma \left(\sqrt{y}\right)\;e^{-my/T}\;,$$

where *T* is the temperature. This relation guarantees that the long-time limit solution of the FPE is the Maxwellian distribution, $e^{-mv^{2}/\left(2T\right)}$. It follows from Equation (25) that g(v) also exponentially decreases with the particle energy as

(26) $$g\left(v\right)=\frac{2\gamma_{0}T\;e^{-v^{2}/u^{2}}}{1+T/(mu^{2})}\;,$$

The model in (24) and (26) can be viewed as the minimal one-dimensional model of surface diffusion of adatoms. The rationale behind the decreasing character of the functions γ(v) and g(v) is that the efficiency of energy exchange between the adatom and the surface should decrease with the adatom’s velocity due to the finite reaction time of the surface atoms. In a previous study [7], a family of the damping functions γ(v) that decrease with kinetic energy was considered, and it was established that all such functions result in a finite diffusion coefficient at all temperatures. The only exception was the function γ(v) from Equation (24), for which the diffusion coefficient tends to be [7]

(27) $$D_{\infty }=\lim_{t\to \infty }\frac{x^{2}\left(t\right)}{2t}=\frac{um}{\gamma_{0}}\left(1+\frac{T}{mu^{2}}\right)\sqrt{\frac{2T}{\pi m}}\int_{0}^{\infty }dy,e^{-y}\int_{0}^{u^{-1}\sqrt{2Ty/m}}dz,e^{z^{2}},$$

which diverges when the temperature exceeds the critical temperature [7]

(28) $$T_{c}=\frac{mu^{2}}{2}\;.$$

For $T>T_{c}$, the particle undergoes superdiffusive motion with an infinite diffusion coefficient $D_{\infty }$.

The superdiffusion of adatoms on solid surfaces, along with the temperature-driven transition from normal to superdiffusive regimes, have been observed in molecular dynamics simulations [22,23]. Although molecular dynamics is numerically precise, it is well known for being a computationally expensive technique. Stochastic simulations offer a suitable alternative. Indeed, the anomalous diffusion of adatoms was reported in simulations of a Langevin equation with constant damping in Ref. [3] but only as a transient process; in the long-time limit, a particle with a constant friction coefficient consistently undergoes normal diffusion [3]. In contrast, the model described by Equations (24) and (26) at $T>T_{c}$ exhibits genuine superdiffusive behavior that persists across all time scales.

### 4.2. Validation of the Numerical Scheme

In our analysis of the model in (24) and (26), we will measure mass in the units of *m*, time in the units of $m/\gamma_{0}$, and length in the units of $mu/\gamma_{0}$. In these units, we have

(29) $$m=\gamma_{0}=u=1\;.$$

Since the first Equation (24) does not involve the position *x*, we can identify the state variable with the velocity, i.e., z≡v. The functions that appear in the update rules (3) and (9) are

(30) $$G\left(v\right)=\frac{T}{1+T}\;e^{-v^{2}}\;,\;\;H\left(v\right)=-\frac{1+2T}{1+T}\;v\;e^{-v^{2}}\;.$$

We compare the standard and the new numerical schemes by calculating the average kinetic energy of the particle in thermal equilibrium for different values of the simulation time step. According to the equipartition theorem, the second moment of the velocity must converge to the value $\langle v^{2}\rangle \to T$ for sufficiently small Δt. In the calculations, we set T=1.

**Figure 1:**
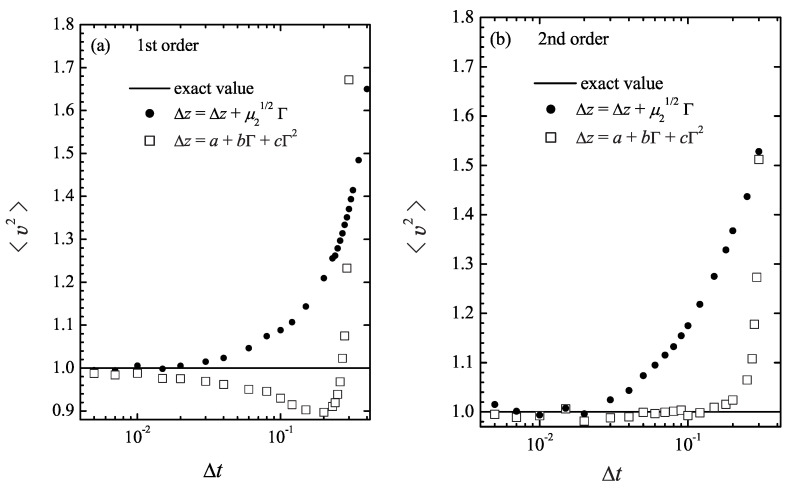
The second velocity moment obtained by simulating the model in (24) and (26) with different time step values Δt at T=1. Panel (**a**) shows the results of the simulations accurate to the first order in Δt, i.e., with terms proportional to $\Delta t^{2}$ removed from Equations (4), (7) and (13). Panel (**b**) shows the values obtained with the second-order accurate simulations. The standard Gaussian simulations, shown as filled circles, are performed according to the rule (3). The results obtained according to the new update rule (9) are shown as open squares.

Figure 1a presents the results of the first-order version of both numerical schemes, in which the terms of order $\Delta t^{2}$ and higher are dropped from the expressions (4), (7), and (13), (23). It can be observed that the values obtained using the standard Gaussian integration scheme (filled circles) slightly overestimate the second moment of velocity for small but finite Δt, while the new scheme (open squares) slightly underestimates it by approximately the same amount. This systematic error may signal that, because of the inaccuracy in the coefficients *a*, *b*, and *c* in the first order, the update rule (9) requires terms of the higher order in Δt to be useful. As the time step decreases below Δt=0.02, both numerical schemes converge to the correct value of $\langle v^{2}\rangle =1$. Overall, the linear versions of both methods demonstrate similar accuracy, so taking skewness into account does not offer any advantages in the first order.

The accuracy of the standard simulation method does not change drastically when the second-order terms in Δt are included, as shown in Figure 1b (solid circles). Similar to its linear version, the standard second-order scheme from Section 2 converges to the correct value at a time step of Δt≤0.02. This confirms the observation made in [10] that the inclusion of the terms of the higher order in the time step does not necessarily improve the accuracy of the calculations.

In contrast, our new integration scheme in the second order becomes accurate at a much bigger time step Δt=0.2 for the parameter values tested, see Figure 1b (open squares). It requires an order of magnitude less computational effort to achieve the same accuracy as the standard numerical scheme. It is interesting to note that at time steps where both schemes fail (Δt>0.02 for the standard and Δt>0.2 for the new one), the error of the new integrator (9) increases with the step size Δt much more rapidly than that of the standard integrator. However, this should not be viewed as a drawback. Indeed, in the simulations of any model, several preliminary trials at varying time steps are always needed in order to determine the optimal Δt value. The significantly higher sensitivity of $\langle v^{2}\rangle$ to Δt observed with the scheme (9), compared to the standard scheme, suggests that selecting the optimal time step should be more straightforward with the new Langevin simulation method.

### 4.3. Time-Dependent Diffusion Coefficient

Next, we apply the scheme from Section 3 to further investigate the model (24)–(29) at different temperatures. Specifically, we focus on the time-dependent diffusion coefficient, which can be expressed in several equivalent forms:

(31) $$D_{t}=\frac{\langle x^{2}\left(t\right)\rangle }{2t}=\int_{0}^{t}dt^{\prime }\;\langle v\left(0\right)\;v\left(t^{\prime }\right)\rangle =\langle v\;{\bar{x}}_{t}\left(v\right)\rangle \;.$$

Here, the initial position is set to x(0)=0, and ${\bar{x}}_{t}\left(v\right)$ is the average distance traveled by the Brownian particle after time *t* given that its velocity at t=0 was *v*. The averaging in the last expression (31) is performed with Maxwellian velocity distribution.

**Figure 2:**
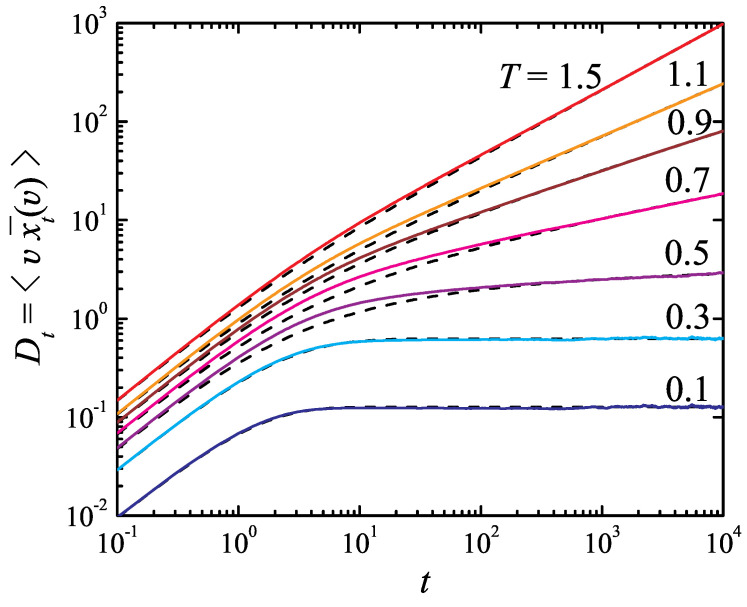
The time-dependent diffusion coefficient for the model (24), (26) at temperatures *T* = 0.1, 0.3, 0.5, 0.7, 0.9, 1.1, and 1.5. Solid colored lines: simulation results obtained by averaging over $10^{6}$ trajectories. Dashed black lines: fitting with the expression (32).

The saturation of the time-dependent diffusion coefficient at $T<T_{c}$ and its power-law increase at $T>T_{c}$ is indeed observed in the simulations; see solid colored curves in Figure 2 featuring the $D_{t}$ plots at a few temperatures above and below $T_{c}=1/2$, as well as at $T=T_{c}$. These curves were obtained with the time step Δt=0.1 with averages taken over $10^{6}$ trajectories. Each curve required approximately 3.5 h of computation. In contrast, using the standard numerical method would have demanded a time step about ten times smaller, resulting in 1.5 days of computation per curve to achieve the same level of accuracy.

We propose to describe the $D_{t}$ plots both below and above the critical temperature with a single empirical formula, which is as follows:

(32) $${\tilde{D}}_{t}=\frac{T\;\tau }{\alpha }\;\left({\left(1+\frac{t}{\tau }\right)}^{\alpha }-1\right)\;.$$

This formula contains two fit parameters: the characteristic time τ and the diffusion coefficient exponent α. It has several useful properties. At short times t≪τ, it yields the time-dependent diffusion coefficient ${\tilde{D}}_{t}=Tt$ (up to the terms $\propto {\left(t/\tau \right)}^{2}$), which is the correct behavior in the ballistic diffusion regime. At long times t≫τ, the diffusion coefficient (32) can exhibit two kinds of behavior depending on the exponent α: if α<0, it converges to a finite long-time limit, while for α>0, it increases as ${\tilde{D}}_{t}\propto t^{\alpha }$. In the limit α→0, its increase is logarithmic: $\lim_{\alpha \to 0}{\tilde{D}}_{t}=T\;\tau \;\ln\left(1+t/\tau \right)$.

The results of fitting the simulation curves with Equation (32) are shown in Figure 2 as dashed black lines. It is seen that the fitting formula correctly captures the short- and long-time behavior of the $D_{t}$ curves obtained in the simulations, whereas it may slightly deviate from the numerical results at the intermediate times t∼τ. The closer the temperature is to the critical value, the more noticeable this deviation becomes.

**Figure 3:**
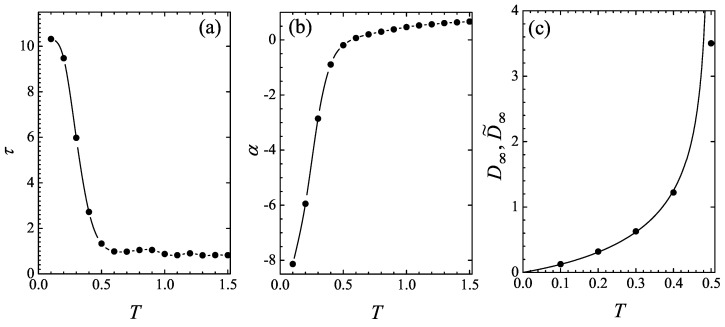
(**a**,**b**) The characteristic time τ and the exponent α from the fit expression (32), respectively; the lines serve to guide the eye. (**c**) The long-time limit of the diffusion coefficient at $T<T_{c}$ as found according to Equation (27) (solid line), and the saturation values of the fit formula (32), namely, ${\tilde{D}}_{\infty }=T\tau /\left|\alpha \right|$ (symbols).

Figure 3 shows the temperature dependence of the parameters obtained by fitting the simulated $D_{t}$ curves with the empirical formula (32). It is seen from Figure 3a that at $T<T_{c}$, the characteristic time strongly decreases with the temperature, while the particle undergoes normal diffusion. In the superdiffusive temperature range $T>T_{c}$, the characteristic time approaches the value τ→1 on heating.

The exponent α vs. temperature plot is shown in Figure 3b. It exhibits a transition from negative values at $T<T_{c}$ to positive values 0<α<1 at $T>T_{c}$ and tends to the ballistic value α→1 as the temperature is increased.

Finally, in the temperature range $T<T_{c}$, in which α<0 (normal diffusion), the long-time value of the diffusion coefficient (32) ${\tilde{D}}_{\infty }=T\tau /\left|\alpha \right|$ should match the theoretical value $D_{\infty }$ from Equation (27). This agreement is indeed seen in Figure 3c at all temperatures tested, except for the critical temperature $T_{c}=1/2$, at which the diffusion coefficient ${\tilde{D}}_{\infty }$ obtained from fitting the simulation curves is large but finite.

Furthermore, if one tried to establish $T_{c}$ as the temperature at which the exponent α goes to zero, then the plot in Figure 3b would suggest the critical temperature value of 0.55, which is 10% higher than the exact result. These inaccuracies are due to the fact that the simulated $D_{t}$-curve at the critical temperature increases logarithmically slowly, implying that a very long time scale needs to be probed to determine the fit parameters in Equation (32) more reliably. This, in turn, would necessitate a much longer simulation time measured in days instead of hours. The standard simulation method, in contrast, would take months instead of days.

## 5. Conclusions

In numerical simulations of the Langevin equation with multiplicative white noise, accounting for the asymmetry in the distribution of the system’s updated state at each time step significantly enhances computational efficiency compared to standard methods that assume a symmetric Gaussian distribution for the updated state. Building on this observation, we have developed a numerical scheme for simulating a univariate Langevin equation with multiplicative Gaussian white noise, which demonstrates superior performance compared to the standard Gaussian simulation method. In our approach, the skewness of the updated state distribution is incorporated into the update rule, which involves adding a second-order polynomial of a Gaussian random number to the state variable.

The improvement in our simulation technique becomes apparent only when the parameters required by the update rule (9) are calculated to the second (or higher) order in the time step Δt. Furthermore, when the white noise is additive, the skewness of the distribution of the updated state variable vanishes, at least in the second order in the time step, and our improved simulation method becomes identical to the standard one.

The high efficiency of this prescription is demonstrated using a specific model of free diffusion, where the damping coefficient decreases exponentially with the kinetic energy of the Brownian particle. This model exhibits a transition from normal diffusion to superdiffusion at a certain critical temperature. A simple empirical formula is introduced to capture the time-dependent diffusion coefficient in both these regimes on all time scales.

While the present paper provides proof-of-principle calculations, the proposed scheme can be extended in several directions. The first one is to generalize it to the multivariate case and to situations where the system is driven by an external time-dependent field. The second improvement would involve incorporating higher-order terms in the time step into the expressions for the moments of the system’s state. Finally, it is plausible that including third and possibly higher powers of the Gaussian random variable in the updating rule (see Equation (9)) could further reduce the computational effort. These possibilities are the focus of our ongoing research.

## Data Availability

No new data were created or analyzed in this study. Data sharing is not applicable to this article.
